# Relationship between lactate-to-albumin ratio and all-cause mortality among critically ill pediatric patients: Results from the pediatric intensive care database

**DOI:** 10.1371/journal.pone.0341727

**Published:** 2026-02-02

**Authors:** Xiangyu Zuo, Yongfu Song, Jun Liu

**Affiliations:** 1 School of Traditional Chinese Medicine, Shanxi Datong University, Datong, China; 2 Department of Pediatrics, The Affiliated Hospital of Changchun University of Chinese Medicine, Changchun, China; 3 Xi’an Maternity and Child Healthcare Hospital, Xi’an, Shaanxi, China; CHUK: Centre Hospitalier Universitaire de Kigali, RWANDA

## Abstract

**Background:**

The lactate-to-albumin ratio (LAR) has emerged as a valuable prognostic marker in adult critically ill patients. However, evidence regarding its association with mortality in pediatric intensive care unit (ICU) patients remains limited. The present study utilized data from the Pediatric Intensive Care (PIC) database to investigate the relationship between LAR and clinical outcomes among critically ill pediatric patients.

**Methods:**

This study enrolled 4,162 pediatric patients from the PIC database as study participants. A piecewise multivariate Cox regression model was constructed, incorporating subgroup analysis and restricted cubic spline (RCS) curve analysis, to investigate the association between the LAR and all-cause mortality during hospitalization and ICU stay.

**Results:**

In the fully adjusted model, for every one-unit increase in LAR, the risk of in-hospital mortality increased by 23% (HR: 1.23, 95% CI: 1.17–1.30), and the risk of ICU mortality increased by 22% (HR: 1.22, 95% CI: 1.15–1.28). The risk of in-hospital mortality and ICU mortality for patients in the highest LAR quartile were 2.30 and 2.17 times those of patients in the lowest quartile, respectively. RCS analysis revealed a non-linear positive correlation between LAR and 30-day all-cause mortality in critically ill children, with a threshold of 0.4468. Moreover, the predictive performance of LAR (AUC = 0.725) was superior to that of lactate alone (AUC = 0.712) or albumin alone (AUC = 0.608).

**Conclusions:**

Research shows that in critically ill pediatric patients, LAR is nonlinearly and positively correlated with the 30-day all-cause mortality rate. Moreover, the higher the LAR value, the more significantly the mortality rate increases, suggesting that it is a promising predictor for short-term mortality risk, although this requires further prospective validation.

## Introduction

Elevated serum lactate levels often indicate tissue hypoxia and hemodynamic disorders [1], and also serve as an indicator of insufficient tissue perfusion and cellular metabolic dysfunction. They are commonly used to assess the severity of illness in patients with shock and multiple organ dysfunction syndrome [2]. Elevated serum lactate is significantly associated with increased mortality in both pediatric and adult patients [2–4]. Serum albumin, synthesized by the liver, is an abundant blood protein and possesses antioxidant and anti-inflammatory properties [5]. Its primary functions include maintaining colloid osmotic pressure in plasma and transporting various substances. Studies have demonstrated that low albumin levels are associated with higher mortality rates, longer hospital stays, and a greater incidence of complications [6–8]. In critical illness, the reduction in serum albumin levels is often due to decreased liver synthesis, enhanced protein catabolism, and increased capillary leakage [9]. Serum albumin serves as an important biomarker for prognosis in intensive care unit (ICU) patients [6–8]. However, when lactate and albumin are used as individual markers, they are susceptible to interference from various factors such as physical activity, liver and kidney function, medication, and nutritional status [10,11].

In critically ill patients with inflammation and tissue hypoxia, serum albumin and lactate levels may exhibit discordant changes [2]. The lactate-albumin ratio (LAR), as a combined indicator of lactate and albumin, has garnered considerable attention for its significant role in the prognosis assessment of critically ill patients. Current research examining the association between LAR and critical illness has predominantly focused on conditions including acute kidney injury, sepsis, acute pancreatitis, and chronic obstructive pulmonary disease [12–15]. Notably, all these studies have focused on adults, while research on critically ill children remains relatively limited. However, despite its physiological plausibility, the prognostic value of LAR in the heterogeneous and physiologically distinct pediatric critical care population has remained inadequately explored. To address this gap, we utilized the PIC database to examine the association between LAR and the risk of in-hospital or ICU mortality among pediatric critically ill patients.

## Methods

### Study design, data source, and population

The population data for this study were obtained from the PIC database, a publicly accessible, single-center retrospective electronic medical record system developed by the Children’s Hospital of Zhejiang University School of Medicine. Detailed information about the database can be accessed via its official website (http://pic.nbscn.org). The PIC database includes clinical records of 12,881 pediatric patients admitted to the hospital’s intensive care unit (ICU) between 2010 and 2018, encompassing data on laboratory tests, medication prescriptions, diagnostic assessments, physical examinations, surgical interventions, and dates of admission and discharge. This study has been approved by the Ethics Committee of the Children’s Hospital Affiliated to Zhejiang University School of Medicine (2019-IRB-052). Given that this study is a retrospective analysis and all patient clinical data have been de-identified, the requirement for informed consent has been waived in accordance with relevant ethical guidelines.

**Fig 1:**
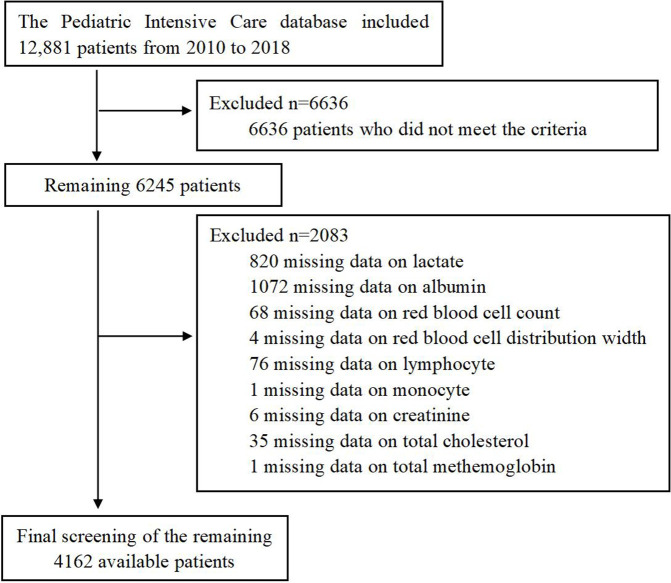
The flow chart of participant selection.

This study included all 12,881 patients from the PIC database. The inclusion criteria were as follows: (a) availability of lactate and albumin data required for LAR calculation; (b) hospitalization duration of at least one day; and (c) complete covariate information. After applying these criteria, a total of 4,162 pediatric patients were included in the final analysis. The selection process and flow of participants are illustrated in Fig 1.

### Variables

All clinical diagnoses were confirmed based on the International Classification of Diseases, Tenth Revision (ICD-10) coding system. The study variables encompassed demographic characteristics (gender, age), length of hospital stay (LOS), length of ICU stay (LOS ICU), medication use (vasopressors, cephalosporins), comorbid conditions (sepsis, heart disease, and pneumonia), as well as laboratory parameters.

All laboratory measurements were obtained within 24 hours of admission. In instances where multiple test results were available within this timeframe, the mean value was utilized for analysis.

### Exposure and outcomes

The LAR was calculated by dividing lactate levels by albumin levels. The primary outcome of this study was 30-day all-cause mortality following hospital admission, while the secondary outcome was 30-day all-cause mortality specifically during ICU stay. For both outcomes, patients were followed from admission until death, hospital discharge, or the 30th day post-admission, whichever occurred first.

### Statistical analysis

Descriptive statistics were performed to summarize the characteristics of the study participants. Continuous variables were summarized using either the mean ± standard deviation (SD) or the median with interquartile range (IQR), depending on their distribution patterns. Categorical variables were described as frequencies and percentages (%). Statistical comparisons between groups were conducted using chi-square tests for categorical variables and t-tests for continuous variables.

The association between the LAR and all-cause mortality was evaluated using both univariate and multivariate Cox proportional hazards models. Model 1 included no adjustment variables. Model 2 was adjusted for sex, age, red blood cell count, platelet count, red blood cell distribution width, lymphocyte count, monocyte count, total protein, chloride, sodium, potassium, calcium, partial pressure of oxygen, and partial pressure of carbon dioxide. Model 3 further incorporated adjustments for total cholesterol, creatinine, uric acid, urea, cystatin C, phosphate, methemoglobin, vasopressors, cephalosporins, heart disease, pneumonia, and sepsis, building upon the covariates included in Model 2. A dose-response relationship between the LAR and all-cause mortality was further assessed using restricted cubic spline (RCS) analysis.

The area under the curve was calculated using receiver operating characteristic (ROC) analysis to evaluate the predictive value of LAR for critical illness in children.

Additionally, subgroup analyses were performed stratified by age, sex, sepsis, heart disease, pneumonia, and the use of vasopressors and cephalosporins to evaluate the consistency of the association between the LAR and all-cause mortality. Statistical significance was assessed by comparing the adjusted hazard ratio (HR) with 1.0 and examining the corresponding 95% confidence interval (CI). All statistical analyses were conducted using R software (version 4.3, http://www.r-project.org/), and a two-tailed *P* < 0.05 was considered statistically significant.

## Results

### Study population and baseline characteristic

In this study, we conducted a comprehensive analysis of the baseline characteristics of 4,162 patients, who were stratified based on the quartiles of LAR (Table 1). Among all patients, 1,815 were female (43.6%) and 2374 were male (56.4%). In terms of laboratory parameters, we observed that the levels of red blood cell distribution width, sodium, potassium, lactate, creatinine, uric acid, phosphate were highest in the Q4 group. In contrast, red blood cell count, platelet count, albumin, total protein, calcium, partial pressure of carbon dioxide, partial pressure of oxygen, and total cholesterol were lowest in the same group.

**Table 1:** Study population characteristics stratified.

Variables	LAR (quartiles)					
	Total	Q1	Q2	Q3	Q4	*P*-value
Gender, n (%)		(≤0.34178)	(0.34189-0.44706)	(0.44729-0.63492)	(≥0.63527)	0.398
Male	2347 (56.4)	600 (57.6)	574 (55.2)	571 (54.9)	602 (57.8)	
Female	1815 (43.6)	441 (42.4)	465 (44.8)	469 (45.1)	440 (42.2)	
Age, Median (IQR)	1.0 (0.0, 4.0)	2.0 (1.0, 4.0)	1.0(0.0, 3.0)	1.0 (0.0, 4.0)	1.0 (0.0, 6.0)	< 0.001
RBC, Mean ± SD	3.8 ± 0.7	3.9 ± 0.7	3.8 ± 0.6	3.8 ± 0.7	3.7 ± 0.9	< 0.001
PLT, Mean ± SD	254.4 ± 138.7	280.1 ± 131.9	259.5 ± 128.5	246.2 ± 135.0	232.0 ± 153.6	< 0.001
RDW, Mean ± SD	14.4 ± 2.4	14.0 ± 2.2	14.3 ± 2.2	14.5 ± 2.4	14.9 ± 2.6	< 0.001
MetHb, Mean ± SD	0.9 ± 0.5	0.9 ± 0.4	0.9 ± 0.6	0.9 ± 0.3	0.9 ± 0.7	0.527
LY, Median (IQR)	2.0 (1.3, 3.1)	2.0 (1.4, 3.0)	2.0 (1.4, 2.7)	2.1 (1.4, 3.0)	2.1 (1.2, 3.7)	0.050
MONO, Median (IQR)	0.6 (0.4, 0.8)	0.5 (0.4, 0.8)	0.6 (0.4, 0.8)	0.6 (0.4, 0.9)	0.6 (0.3, 0.9)	< 0.001
Albumin, Mean ± SD	37.5 ± 6.3	39.7 ± 5.2	38.6 ± 5.3	37.4 ± 5.6	34.2 ± 7.4	< 0.001
TP, Mean ± SD	57.4 ± 9.6	60.7 ± 8.7	58.3 ± 8.2	57.0 ± 9.2	53.6 ± 10.7	< 0.001
Na, Mean ± SD	137.6 ± 5.0	137.2 ± 4.1	137.5 ± 4.1	137.7 ± 4.4	138.0 ± 6.8	0.002
K, Mean ± SD	3.8 ± 0.5	3.7 ± 0.4	3.8 ± 0.4	3.7 ± 0.5	3.8 ± 0.6	0.043
Ca, Mean ± SD	1.2 ± 0.1	1.2 ± 0.1	1.2 ± 0.1	1.2 ± 0.1	1.2 ± 0.1	< 0.001
Chloride, Mean ± SD	108.1 ± 5.6	108.1 ± 5.4	108.1 ± 5.0	108.2 ± 5.0	108.1 ± 6.9	0.953
PaCO₂, Mean ± SD	38.3 ± 8.9	38.5 ± 9.8	38.7 ± 7.8	38.3 ± 7.9	37.7 ± 9.7	0.082
PaO₂, Mean ± SD	147.2 ± 49.7	147.8 ± 48.1	155.0 ± 49.7	148.9 ± 49.9	137.0 ± 49.4	< 0.001
Lactate, Median (IQR)	1.7 (1.3, 2.3)	1.1 (0.9, 1.2)	1.5 (1.4, 1.7)	2.0 (1.7, 2.2)	3.1 (2.5, 4.1)	< 0.001
TC, Mean ± SD	3.0 ± 1.3	3.4 ± 1.1	3.0 ± 1.2	2.9 ± 1.2	2.8 ± 1.4	< 0.001
Cr, Median (IQR)	41.5 (35.5, 50.0)	39.0 (33.0, 46.5)	40.0 (35.0, 46.0)	41.5 (36.5, 49.0)	47.2 (38.9, 60.2)	< 0.001
Urea, Median (IQR)	3.6 (2.6, 4.8)	3.3 (2.5, 4.4)	3.5 (2.6, 4.4)	3.6 (2.7, 4.7)	4.0 (2.9, 6.0)	< 0.001
Uric Acid, Mean ± SD	306.0 ± 177.3	284.6 ± 146.3	286.7 ± 132.9	297.8 ± 170.7	354.6 ± 233.1	< 0.001
Cys C, Mean ± SD	1.0 ± 0.6	0.9 ± 0.5	0.9 ± 0.4	1.0 ± 0.7	1.1 ± 0.6	< 0.001
Phosphate, Mean ± SD	1.6 ± 0.6	1.5 ± 0.5	1.5 ± 0.4	1.6 ± 0.5	1.7 ± 0.8	< 0.001
LOS hospital, Median (IQR)	12.9 (7.0, 20.1)	10.8 (6.1, 16.1)	13.0 (8.0, 19.2)	14.0 (8.1, 21.7)	13.0 (6.0, 23.7)	< 0.001
LOS ICU, Mean ± SD	3.9 (2.0, 8.5)	3.8 (1.8, 8.2)	3.7 (1.9, 6.8)	3.9 (2.0, 7.4)	5.6 (2.6, 12.0)	< 0.001
Vasopressors, n (%)						0.14
No	2738 (65.8)	706 (67.8)	662 (63.7)	698 (67.1)	672 (64.5)	
Yes	1424 (34.2)	335 (32.2)	377 (36.3)	342 (32.9)	370 (35.5)	
Cephalosporins, n (%)						0.891
No	2699 (64.8)	674 (64.7)	666 (64.1)	674 (64.8)	685 (65.7)	
Yes	1463 (35.2)	367 (35.3)	373 (35.9)	366 (35.2)	357 (34.3)	
pneumonia, n (%)						0.604
No	3700 (88.9)	924 (88.8)	932 (89.7)	928 (89.2)	916 (87.9)	
Yes	462 (11.1)	117 (11.2)	107 (10.3)	112 (10.8)	126 (12.1)	
Heart disease, n (%)						< 0.001
No	3813 (91.6)	988 (94.9)	920 (88.5)	934 (89.8)	971 (93.2)	
Yes	349 (8.4)	53 (5.1)	119 (11.5)	106 (10.2)	71 (6.8)	
Sepsis, n (%)						< 0.001
No	4029 (96.8)	1024 (98.4)	1013 (97.5)	1013 (97.4)	979 (94)	
Yes	133 (3.2)	17 (1.6)	26 (2.5)	27 (2.6)	63 [6]	

Notes: RBC: red blood cell count, PLT: platelet count, RDW: red blood cell distribution width, MetHb: methemoglobin, LY: lymphocyte, MONO: monocyte, TP: total protein; Na: sodium; K: potassium; Ca: calcium; PaCO₂: partial pressure of carbon dioxide; PaO₂: partial pressure of oxygen; TC: total cholesterol; Cr: creatinine; Cys C: Cystatin C.

### The relationship between LAR and 30-day mortality rate

When LAR was treated as a continuous variable, analysis under the fully adjusted Model 3 revealed that each one-unit increase in LAR was associated with a 23% higher risk of in-hospital mortality among pediatric patients (HR: 1.23, 95% CI: 1.17–1.30, *P* < 0.001) and a 22% higher risk of ICU mortality (HR: 1.22, 95% CI: 1.15–1.28, *P* < 0.001) (Table 2).

**Table 2:** Multivariate analyses of variables for 30-day mortality.

Variable	Model 1	*P-value*	Model 2	*P-value*	Model 3	*P-value*
	HR (95%CI)		HR (95%CI)		HR (95%CI)	
30-day in-hospital mortality	1.31(1.26 ~ 1.36)	<0.001	1.2(1.14 ~ 1.26)	<0.001	1.23(1.17 ~ 1.30)	<0.001
Q1(≤0.34178)	1(Ref)		1(Ref)		1(Ref)	
Q2(0.34189 ~ 0.44706)	0.86(0.51 ~ 1.44)	0.56	0.92(0.54 ~ 1.55)	0.746	0.87(0.51 ~ 1.46)	0.588
Q3(0.44729 ~ 0.63492)	0.94(0.57 ~ 1.55)	0.796	0.92(0.55 ~ 1.53)	0.74	0.90(0.54 ~ 1.51)	0.698
Q4(≥0.63527)	4.14(2.77 ~ 6.19)	<0.001	2.66(1.72 ~ 4.11)	<0.001	2.3(1.48 ~ 3.59)	<0.001
Trend	1.86(1.62 ~ 2.14)	<0.001	1.49(1.29 ~ 1.72)	<0.001	1.41(1.21 ~ 1.64)	<0.001
30-day in-ICU mortality	1.30 (1.25 ~ 1.35)	<0.001	1.2(1.14 ~ 1.26)	<0.001	1.22(1.15 ~ 1.28)	<0.001
Q1(≤0.34178)	1(Ref)		1(Ref)		1(Ref)	
Q2(0.34189 ~ 0.44706)	1.06(0.64 ~ 1.78)	0.811	1.1(0.66 ~ 1.85)	0.718	1(0.59 ~ 1.68)	0.99
Q3(0.44729 ~ 0.63492)	1.03(0.62 ~ 1.7)	0.907	1.02(0.61 ~ 1.7)	0.933	0.97(0.58 ~ 1.61)	0.896
Q4(≥0.63527)	3.43(2.3 ~ 5.12)	<0.001	2.64(1.71 ~ 4.07)	<0.001	2.17(1.39 ~ 3.37)	<0.001
Trend	1.64(1.43 ~ 1.87)	<0.001	1.44(1.25 ~ 1.66)	<0.001	1.35(1.17 ~ 1.56)	<0.001

Model 1: no adjusted.

Model 2: adjusted for gender, age, RBC, PLT, RDW, LY, MONO, TC, chloride, Na, K, Ca, PaO2, PaCO2.

Model 3: Model 2 + TC, Cr, uric acid, urea, Cys C, phosphate, methemoglobin, vasopressors, cephalosporins, heart disease, pneumonia, and sepsis.

When LAR was used as a stratifying variable, the fully adjusted Model 3 revealed that, with the Q1 group as the reference, the Q2 and Q3 groups exhibited no statistically significant association with in-hospital mortality (*P* > 0.05). In contrast, children in the Q4 group exhibited a significantly increased risk of in-hospital mortality (HR: 2.30, 95% CI: 1.48–3.59, *P* < 0.001). In the analysis of the secondary outcome, the Q2 and Q3 groups continued to exhibit no statistically significant association with mortality risk (*P* > 0.05). In contrast, the Q4 group demonstrated a significantly elevated risk of death in the pediatric ICU (HR: 2.17, 95% CI: 1.39–3.37, *P* < 0.001) (Table 2).

**Fig 2:**
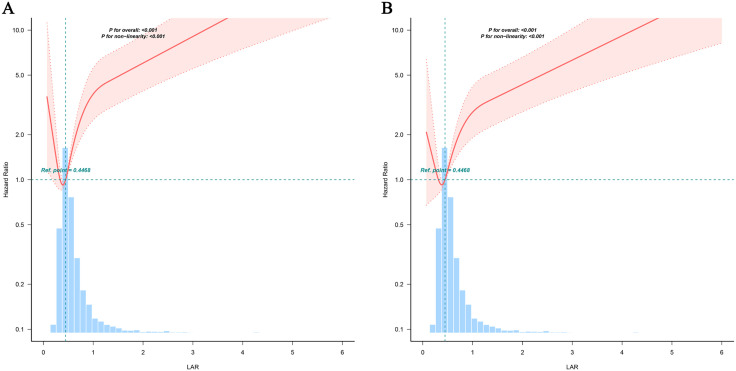
Association between LAR and the risk of 30-day mortality based on restricted cubic spline curves. (A) 30-day in hospital mortality. (B)30-day in ICU mortality. Adjusted for gender, age, RBC, PLT, RDW, LY, MONO, TC, chloride, Na, K, Ca, PaO2, PaCO2, TC, Cr, uric acid, urea, Cys C, phosphate, methemoglobin, vasopressors, cephalosporins, heart disease, pneumonia, and sepsis.

After adjusting for key confounding factors, RCS analysis demonstrated a statistically significant nonlinear association between LAR and all-cause mortality among pediatric patients, with a threshold value of 0.4468 (all *P* < 0.05, Fig 2).

**Fig 3:**
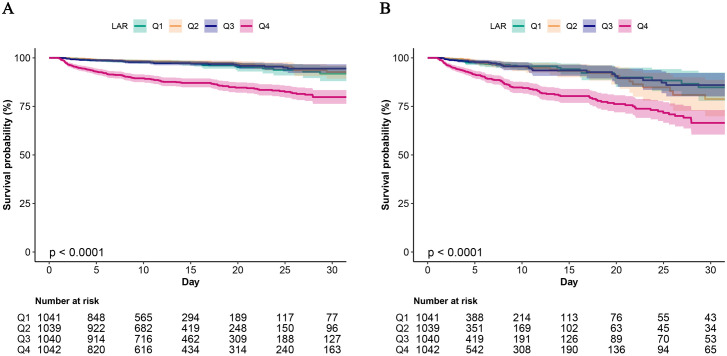
Kaplan-Meier analysis for 30-day survival probability in critically ill pediatric patients. (A) Kaplan-Meier analysis of in-hospital mortality within 30 days. (B) Kaplan-Meier analysis of ICU mortality within 30 days.

Fig 3 shows the Kaplan-Meier analysis, depicting the cumulative 30-day mortality risk. The all-cause mortality risk in the Q4 group was significantly higher than those in the Q1-Q3 groups.

**Fig 4:**
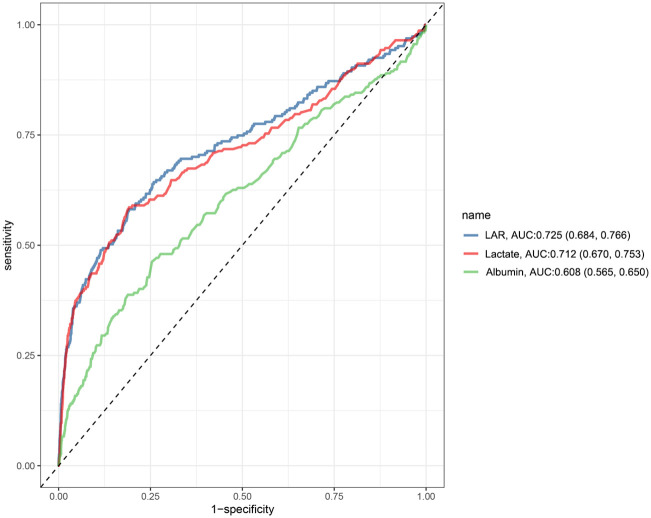
Receiver operating characteristic curves of LAR, lactate, and albumin for predicting in-ICU mortality.

The predictive performance of lactate, albumin, and LAR for all-cause mortality in critically ill pediatric was evaluated using receiver operating characteristic (ROC) curve analysis. As illustrated in Fig 4, LAR demonstrated the highest AUC value of 0.725, followed by lactate with an AUC of 0.712, while albumin exhibited the lowest AUC of 0.608. These results suggest that LAR provides superior predictive accuracy for mortality risk in critically ill children compared to either lactate or albumin alone.

### Subgroup analyses

**Fig 5:**
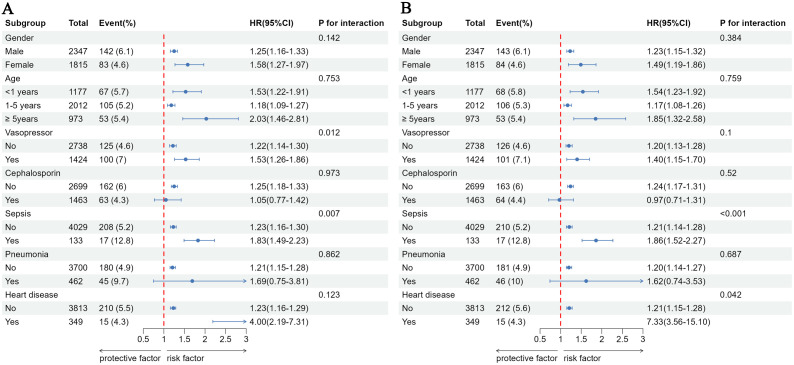
Association between LAR and the risk of 30-day mortality in different subgroups. (A) 30-day in hospital mortality. (B) 30-day in ICU mortality. Adjusted for gender, age, RBC, PLT, RDW, LY, MONO, TC, chloride, Na, K, Ca, PaO2, PaCO2, TC, Cr, uric acid, urea, Cys C, phosphate, methemoglobin, vasopressors, cephalosporins, heart disease, pneumonia, and sepsis.

Fig 5 presents the results of the subgroup analysis, demonstrating that no significant interactions were observed for age, gender, pneumonia, or cephalosporin use (*P* > 0.05). There was an interaction in both primary and secondary outcomes for pediatrics with sepsis (*P* < 0.05). In the primary outcome, there was an interaction in the use of vasopressors, but not in the secondary outcome. A history of heart disease had an interaction in the secondary outcome, but not in the primary outcome. Heart disease had the greatest impact on in-hospital mortality (HR: 4.00, 95% CI: 2.19–7.31), and remained the most significant in secondary outcomes (HR: 7.33, 95% CI: 3.56–15.1).

### Sensitivity analyses

We conducted two sensitivity analyses to evaluate the robustness of the association between LAR and all-cause mortality. In the first sensitivity analysis, multiple imputation was employed to address missing data. The results demonstrated that the association between LAR and in-hospital mortality remained consistent (HR: 1.23, 95% CI: 1.17–1.30, *P* < 0.001). Similarly, for the secondary outcome, the relationship between LAR and ICU mortality was also stable (HR: 1.22, 95% CI: 1.15–1.28, *P* < 0.001) (S1 Table 1). In the second sensitivity analysis, we excluded patients with heart disease comorbidity. We found that the relationship between LAR and in-hospital mortality remained stable (HR: 1.23, 95% CI: 1.17–1.30, *P* < 0.001), and the association also remained stable for the secondary outcome (HR: 1.22, 95% CI: 1.15–1.28, *P* < 0.001) (S2 Table).

## Discussion

This large single-center cohort study enrolled 4,162 critically ill pediatric patients from the PIC database. After adjusting for multiple potential confounders, a non-linear association was identified between the LAR and both 30-day all-cause in-hospital and ICU mortality, with an inflection point at 0.4468. Our findings demonstrate that elevated LAR levels are associated with increased mortality risk. Notably, the highest LAR quartile (≥0.63527) emerged as a significant independent risk factor for both in-hospital and ICU mortality. Based on comprehensive analysis, our results indicate that the LAR is an independent predictor of 30-day all-cause mortality in critically ill pediatric patients. These findings suggest that the LAR is a promising and independent indicator for assessing short-term clinical prognosis in critically ill children. Its association with mortality risk, especially beyond the identified threshold, indicates that it may help in risk stratification. Future prospective, multi-center studies are warranted to validate its clinical utility before it can be recommended for routine emphasis at admission.

A Japanese study on adult critical care demonstrated that LAR may serve as a useful predictor for ICU mortality, with an AUC of 0.761 (95% CI 0.757–0.765) [16]. Another study focusing on pediatric populations indicated that LAR is a reliable prognostic indicator for in-hospital mortality among critically ill pediatric patients, with an AUC of 0.737 [17]. Consistent with the findings of the aforementioned studies, our results demonstrate that LAR serves as an independent risk factor for both in-hospital and ICU mortality among pediatric critically ill pediatric patients, with an AUC of 0.75, which is superior to the predictive performance of lactate or albumin alone.

Studies have shown that hyperlactatemia serves as an independent risk factor for mortality among critically ill adults and children [3,4]. Lactate is a product of anaerobic metabolism and is predominantly produced via the glycolytic pathway during systemic hypoxia [18]. Elevated levels of lactate serve as an indicator of tissue hypoxia, metabolic disturbances, and systemic inflammatory responses [19]. A meta-analysis emphasizes that in the treatment of critical diseases such as sepsis, there is a need to dynamically monitor lactate levels and evaluate its clearance rate [20]. Impairment of hepatic or renal function in patients may lead to lactate accumulation, thereby increasing its systemic concentrations [21]. In addition, the administration of certain medications, such as metformin or salbutamol, may lead to abnormal elevations in lactate levels [22]. Serum albumin, as an acute-phase reactant, reflects both the inflammatory status and systemic nutritional condition of the body [14]. In patients with sepsis, its association with prognosis has been validated in studies [23]. Hypoalbuminemia may be associated with various clinical conditions, including chronic malnutrition, persistent inflammatory states, and hepatic dysfunction [2]. However, the levels of lactate and albumin are influenced by multiple factors, which limits their predictive accuracy when used as standalone biomarkers. As a composite indicator, LAR offers more comprehensive insights into patient prognosis. It demonstrates notable advantages in predicting mortality, complication rates, and other prognostic outcomes in critically ill patients [5]. Elevated LAR levels are consistently associated with poorer clinical outcomes, highlighting its role as a prognostic marker with high sensitivity and specificity [2]. For critically ill pediatric patients, early assessment of their LAR can facilitate more effective risk prevention and control and provide guidance for guiding the intensity of therapeutic interventions. In addition, clarifying the key threshold of LAR can serve as a basis for developing targeted treatment plans, thereby reducing the risk of adverse prognosis and mortality in patients.

In the subgroup analysis, we observed that LAR exerted a significant impact on critically ill pediatric patients with cardiac conditions. Studies have demonstrated that in patients with acute coronary syndrome, elevated serum lactate levels and decreased albumin levels are both associated with poor prognosis and increased mortality [8,24,25]. Elevated lactate levels also serve as a significant prognostic marker in patients with heart failure, indicating a markedly increased risk of mortality [7,26]. The serum lactate and albumin levels of patients with cardiogenic shock are also closely associated with the severity of the condition and prognosis [27,28]. LAR exhibits effective predictive value for cardiovascular disease mortality. Elevated LAR levels have been established as an independent risk factor for increased mortality among patients with severe heart failure and coronary heart disease [5,29,30].

Patients with heart disease often have impaired blood perfusion, which leads to diminished oxygen delivery to the myocardium and a shift to anaerobic metabolism. The by-product, lactate, accumulates and is released into the systemic circulation during ischemia. Coupled with the decreased ability of the myocardium to clear lactate, endothelial dysfunction, and sympathetic nervous system activation, these factors collectively contribute to elevated lactate levels [5]. At the same time, in patients with heart disease, albumin synthesis may be reduced and its levels reduced due to hepatic dysfunction, insufficient nutrient intake, or hepatorenal dysfunction caused by heart failure. Chronic diuretic use in patients with heart disease can also cause tissue hypoperfusion and anaerobic metabolism [29].

This study also has several limitations. Firstly, this study is a retrospective study, and the causal relationship between LAR and all-cause mortality cannot be inferred. Secondly, this study included only patients from a pediatric hospital in Zhejiang Province, China. Therefore, the generalizability of the research results is unclear. Thirdly, due to data limitations, other potential confounding variables were not included, which may have exerted some influence on the final results. Furthermore, as this is a single-center study, our findings require validation in large-scale, multi-center, prospective cohorts to confirm the generalizability of LAR as a robust prognostic tool across diverse PICU populations and clinical settings.

## Conclusion

This study demonstrated that LAR is an important risk factor for all-cause mortality in critically ill children, and it exhibits a non-linear association with the 30-day in-hospital and ICU all-cause mortality, with a threshold of 0.4468. Moreover, a higher LAR value is associated with an increased mortality risk. These findings suggest that the LAR is a promising and independent indicator for assessing short-term clinical outcomes in critically ill pediatric patients. Its association with mortality risk, particularly beyond a defined threshold, supports its potential role in risk stratification. Future prospective, multi-center studies are warranted to validate its clinical utility and to establish its role in facilitating early mortality prediction in the PICU before it can be recommended for routine clinical use.

## Supporting information

S1 Table Association between LAR and all-cause mortality after multiple imputation for missing data. (DOCX)

S2 Table Association between LAR and all-cause mortality after excluding patients with heart disease comorbidity. (DOCX)
