# Supplementary material for: Relationship between lactate-to-albumin ratio and all-cause mortality among critically ill pediatric patients: Results from the pediatric intensive care database
Source: PLoS One. 2026 Feb 2;21(2):e0341727. doi: 10.1371/journal.pone.0341727 (PMC12863524; doi:10.1371/journal.pone.0341727)
Supplement: S1 Table — (DOCX) [file pone.0341727.s001.docx]

**Supporting information**

**S1 Table. Association between LAR and all-cause mortality after multiple imputation for missing data.**

| Variable | Model 1 | *P-value* | Model 2 | *P-value* | Model 3 | *P-value* |
| --- | --- | --- | --- | --- | --- | --- |
|  | HR (95%CI) |  | HR (95%CI) |  | HR (95%CI) |  |
| 30-day in-hospital mortality | 1.32(1.27~1.37) | <0.001 | 1.20(1.14~1.26) | <0.001 | 1.23(1.17~1.30) | <0.001 |
| Q1(<0.34091) | 1(Ref) |  | 1(Ref) |  | 1(Ref) |  |
| Q2(0.34097~0.44805) | 0.86(0.52~1.41) | 0.541 | 0.90(0.53~1.52) | 0.696 | 0.85(0.5~1.43) | 0.54 |
| Q3(0.4410~0.64057) | 0.95(0.59~1.53) | 0.826 | 0.90(0.54~1.50) | 0.689 | 0.86(0.52~1.44) | 0.572 |
| Q4(>0.64067) | 4.11(2.8~6.03) | <0.001 | 2.75(1.79~4.24) | <0.001 | 2.36(1.52~3.68) | <0.001 |
| 30-day in-ICU mortality | 1.30 (1.25~1.35) | <0.001 | 1.20(1.15~1.26) | <0.001 | 1.22(1.15~1.28) | <0.001 |
| Q1(<0.34091) | 1(Ref) |  | 1(Ref) |  | 1(Ref) |  |
| Q2(0.34097~0.44805) | 1.09(0.67~1.79) | 0.717 | 1.07(0.64~1.80) | 0.785 | 0.98(0.58~1.66) | 0.951 |
| Q3(0.4410~0.64057) | 1.05(0.65~1.69) | 0.843 | 1.01(0.61~1.67) | 0.979 | 0.93(0.56~1.56) | 0.793 |
| Q4(≥0.64067) | 3.43(2.34~5.03) | <0.001 | 2.69(1.75~4.14) | <0.001 | 2.23(1.43~3.46) | <0.001 |

Model 1: no adjusted.

Model 2: adjusted for gender, age, RBC, PLT, RDW, LY, MONO, TC, chloride, Na, K, Ca, PaO2, PaCO2.

Model 3: Model 2 +TC, Cr, urea, Cys C, phosphate, methemoglobin, vasopressors, cephalosporins, heart disease, pneumonia, and sepsis
