# Supplementary material for: Relationship between lactate-to-albumin ratio and all-cause mortality among critically ill pediatric patients: Results from the pediatric intensive care database
Source: PLoS One. 2026 Feb 2;21(2):e0341727. doi: 10.1371/journal.pone.0341727 (PMC12863524; doi:10.1371/journal.pone.0341727)
Supplement: S2 Table — (DOCX) [file pone.0341727.s002.docx]

**S2 Table. Association between LAR and all-cause mortality after excluding patients with heart disease comorbidity.**

| Variable | Model 1 | *P-value* | Model 2 | *P-value* | Model 3 | *P-value* |
| --- | --- | --- | --- | --- | --- | --- |
|  | HR (95%CI) |  | HR (95%CI) |  | HR (95%CI) |  |
| 30-day in-hospital mortality | 1.31(1.26~1.36) | <0.001 | 1.20(1.14~1.26) | <0.001 | 1.23(1.17~1.30) | <0.001 |
| Q1(≤0.34178) | 1(Ref) |  | 1(Ref) |  | 1(Ref) |  |
| Q2(0.34189~0.44706) | 0.86(0.51~1.44) | 0.56 | 0.91 (0.54~1.53) | 0.723 | 0.86(0.51~1.46) | 0.586 |
| Q3(0.44729~0.63492) | 0.94(0.57~1.55) | 0.796 | 0.91 (0.55~1.51) | 0.715 | 0.9(0.54~1.5) | 0.696 |
| Q4(≥0.63527) | 4.14(2.77~6.19) | <0.001 | 2.59 (1.68~3.99) | <0.001 | 2.30(1.48~3.58) | <0.001 |
| 30-day in-ICU mortality | 1.30 (1.25~1.35) | <0.001 | 1.20(1.15~1.26) | <0.001 | 1.22(1.15~1.28) | <0.001 |
| Q1(<0.34091) | 1(Ref) |  | 1(Ref) |  | 1(Ref) |  |
| Q2(0.34097~0.44805) | 1.06 (0.64~1.78) | 0.811 | 1.09 (0.65~1.83) | 0.749 | 1.01 (0.6~1.7) | 0.965 |
| Q3(0.4410~0.64057) | 1.03 (0.62~1.7) | 0.907 | 1.01 (0.61 ~1.68) | 0.964 | 0.98 (0.59~1.63) | 0.93 |
| Q4(>0.64067) | 3.43 (2.3 ~ 5.12) | <0.001 | 2.57 (1.67~ 3.96) | <0.001 | 2.17 (1.4~3.38) | 0.001 |

Model 1: no adjusted.

Model 2: adjusted for gender, age, RBC, PLT, RDW, LY, MONO, TC, chloride, Na, K, Ca, PaO2, PaCO2.

Model 3: Model 2 +TC, Cr, urea, Cys C, phosphate, methemoglobin, vasopressors, cephalosporins, heart disease, pneumonia, and sepsis
